# EACH Erasmus Mundus programme: advancing excellence in analytical chemistry education and industry impact

**DOI:** 10.1007/s00216-025-05734-1

**Published:** 2025-01-29

**Authors:** Riin Rebane, Anu Teearu, Irja Helm, Johan Bobacka, Jérôme Randon, Jonas Bergquist, Ivo Leito

**Affiliations:** 1 Institute of Chemistry, University of Tartu, Ravila 14a, 50411 Tartu, Estonia; 2 Laboratory of Molecular Science and Engineering, Faculty of Science and Engineering, Åbo Akademi University, Henriksgatan 2, 20500 Turku, Finland; 3 Université Claude Bernard Lyon1, ISA, UMR 5280, CNRS, 5 Rue de La Doua, 69100 Villeurbanne, France; 4 Department of Chemistry – BMC, Analytical Chemistry and Neurochemistry, Uppsala University, Husargatan 3, 75124 Uppsala, Sweden

## Introduction: recap and purpose of this follow-up

In today’s rapidly evolving world, innovation is key to solving pressing challenges such as mitigating climate change, reducing environmental pollution, developing renewable energy and sustainable food sources, improving healthcare and medication, and finding new ways for waste valorisation and life cycle management. Analytical chemistry plays a crucial role in these areas by including industrial process analysis and environmental monitoring, ensuring food quality and authenticity as well as aiding in the development of active pharmaceutical ingredients and medical diagnostics. As a result of the ever-evolving challenges, currently, there are countless exciting and rewarding opportunities for analytical chemists. At the same time, however, the scope of the knowledge and skills required as well as the overall job market has also evolved considerably [1–3].

Higher education institutions have not always been able to keep pace with these developments. Recognising this, around 10 years ago, four renowned European universities in the field of analytical chemistry—University of Tartu (UT), Estonia; Uppsala University (UU), Sweden; Claude Bernard University Lyon 1 (UCBL), France; and Åbo Akademi University (ÅAU), Finland—together with numerous associated partners have set up the master’s programme Excellence in Analytical Chemistry (EACH, https://www.analyticalchemistry.eu). In 2015, the programme welcomed its first cohort of students [4]. The programme was specifically designed to bridge the theoretical and practical knowledge gaps between the classical study programmes of analytical chemistry, newly emerging fields of analytical chemistry, and entrepreneurial problem-solving of modern socio-economic challenges.

**Fig. 1:**
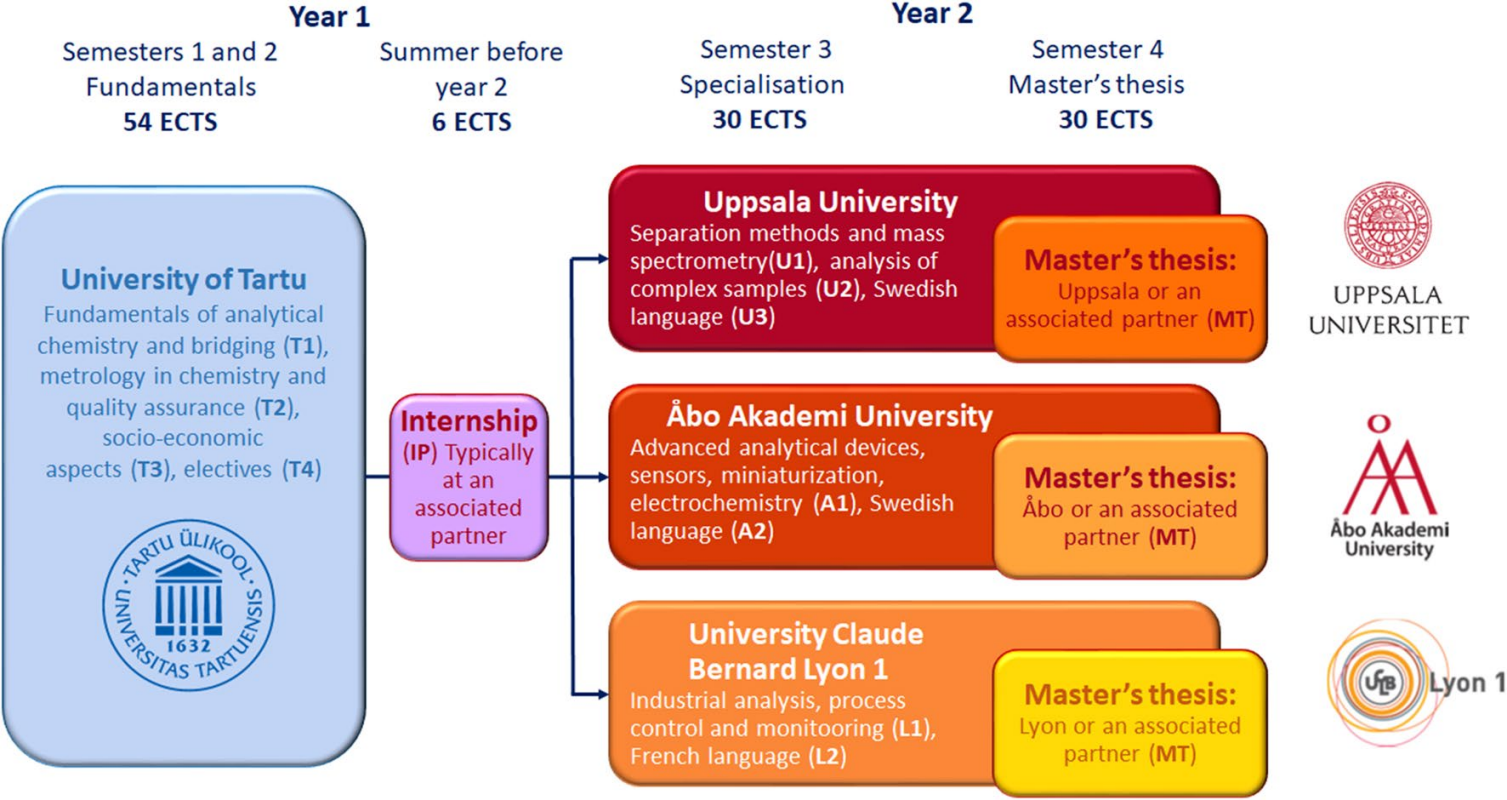
The EACH programme’s structure. Module codes are indicated in parentheses

The EACH programme is run under the guidance of internationally renowned European analytical chemistry educators and practitioners—from participating institutions as well as by visiting scholars—and provides a coherent, compatible, and international learning environment, where mobility is intrinsically embedded into the programme’s structure (see Fig. 1). The complementary nature of EACH partner universities’ strengths and the mandatory mobility of studying at two partner universities (with the possibility of completing the master’s thesis at an associated partner institution or other) combines a comprehensive fundamental analytical chemistry and metrology education with a strong practical application component.

After almost 10 years since the course’s inception, it is time to reflect on how has the EACH programme succeeded in achieving the set goals and to provide an update on recent developments and the programme’s impacts as well as to recognise new trends in analytical chemistry education since the initial report about the EACH programme in 2019 [4].

## Update on the EACH programme

### Current status of the programme

To provide the best overview of EACH programme’s progress, it is important to reiterate that the programme aimed to ensure the acquisition of strong fundamental knowledge of analytical chemistry, measurement science, and novel emerging areas of analytical chemistry as well as applied and practical knowledge of analytical methods. Over the years, the programme has evolved to ensure the compatibility of graduates’ knowledge and practical skills with workforce market and employer expectations.

One of the main focus points of the programme is the relevance of the course content. In addition to high-level analytical chemistry training, socio-economic aspects of analytical chemistry (first-year courses), which are related to transferable (soft) skills and include economic and legal components, have been key components of the programme from the beginning. However, in recent years, this has been expanded even more. As a result, students get a comprehensive picture of the economic and legal impact of analyses on society, direct and indirect costs and benefits, practical management of an analytical laboratory, analyses specified in legislation, requirements for laboratories in terms of competence and accreditation, consequences of non-compliance with legal limits, etc. Additionally, there is a significant number of new elective courses (data handling, entrepreneurship, etc.), many of which have been introduced into the curriculum at the request of students. The learning outcomes of the programme are presented in Table 1.

**Table 1:** Learning outcomes of the EACH programme together with the relevant contributing modules

Competences/learning outcomes	Relevant modules^a^
***Field-specific competencies***	
1. Has a systematic understanding of the physical, chemical and metrological foundations of analytical chemistry; factors affecting analytical results; methods for calculating and presenting results and evaluating the **quality and reliability of the results** for the contemporary and emerging chemical analysis methods	**T1, MT** U1, U2, L1, A1, T4
1.a. Has a good overview of the recent developments and emerging areas of analytical chemistry	**WS**
1.b. Has a good understanding and skills for application of the **main concepts and approaches of metrology** in chemistry	**T2**, MT
2. Has a systematic understanding of quality systems, economic and legal aspects of chemical analysis and a basic understanding of an analytical laboratory management, including **maintenance of a quality management system**	**T2, T3, IP** MT
3. Has the **skills to work** with the variety of analysis and sample preparation techniques and to tune and adjust them according to specific analysis tasks; to optimise analysis procedures; to carry out data evaluation and sampling. Has the background to learn and apply emerging techniques	**T1, U1, U2, L1, A1**, MT
4. **Can define the analytical problem** select appropriate techniques, develop novel methods, test them and determine their characteristics, assess their suitability for the task (validation) and apply corrective actions *in one of the subfields* (4.a–4.c) of analytical chemistry	**MT**, T1, IP
4.a. Separation science and organic analysis (GC, LC, MS, …), including multimodal separation techniques and complex samples of biological importance;	**U1, U2**, T4
4.b. Industrial analytical chemistry and process control;	**L1**
4.c. Electroanalysis, as well as electrochemical and biosensors, including their design, miniaturisation and uses for different analytical tasks	**A1**, T4
5. Has the knowledge and skills to **evaluate the suitability and reliability of the acquired data** and results obtained either by themselves or by others	**T2, MT**, IP
***Generic competences***	
6. Is able to use analytical knowledge and skills for problem-solving and fast decision-making in unfamiliar and complex situations using broad **inter- and multidisciplinary contexts**	**IP, MT**
7. Is able to disseminate and rationalise scientific communication to both—a scientific—including in **inter- and multidisciplinary** settings—and non-scientific audience	**MT, IP**, WS, T2
8. Is able to learn autonomously and use contemporary approaches for information retrieval	**MT, IP**, T2
9. Has entrepreneurial attitude, good management and teamwork skills	**T3, L1, IP**
10. Has basic proficiency in one of the languages spoken in the countries involved in the EACH programme and is familiar with the main cultural characteristics of these countries	**T4, U3, A2, L2,** IP
11. Is proficient in statistical analysis of laboratory data using state-of-the-art software solutions and programs (e.g. R, Python)	**T1, T2, IP, U1, L1, A1, MT**

^a^See Fig. 1 for module definitions. Modules written in bold contribute most to achieving the respective learning outcomes

While running for the last 10 years, the visibility of the EACH programme has been constantly increasing thanks to the suggestions received from our student ambassadors and other stakeholders via the organised feedback system. The EACH programme’s webpage (especially the blog/news section) is regularly updated. Moreover, EACH community maintains a strong presence on social media platforms such as Facebook and LinkedIn. The EACH programme is frequently popularised at student fairs and conferences. Additionally, help and guidance are available for newly admitted students. EACH alumni have a significant role in forming the “EACH community” (EACH network). They participate in promoting the programme and are members of the EACH “dissemination committee”—a group of students and alumni who organise promotion of the programme through various channels. The EACH network provides information exchange on job offers and PhD positions. Alumni also provide guidance on how to obtain a position or act as recruiters/recommenders for the EACH students. As the programme’s recognition grows, a significant rise in the partner offerings of internship placements and available job opportunities for EACH students and alumni has been observed.

### Study tracks

One of the key features of the EACH programme’s study track scheme (Fig. 1) is that the study time is split between two universities (and/or an optional associated partner for the master’s thesis). All students spend their first study year at UT (also referred to as the first-year university) learning the fundamentals of analytical chemistry, quality assurance and metrology in chemistry, and the socio-economic aspects of analytical chemistry. The second study year is specialisation-oriented and is spent either at UU, UCBL, or ÅAU (also referred to as the second-year universities). The second half of the second study year (fourth semester) is typically spent on working towards the master’s thesis either at the respective second-year university or an associated partner institution, such as a professional laboratory or industry. This arrangement makes this programme highly special as students can pursue their future interests by building on a solid unified base of analytical chemistry.

All current EACH students and alumni have expressed satisfaction with this simple and clear study track scheme. In particular, students appreciate starting at the same university and the mobility periods are typically 1 + 1 year, i.e. the programme is not overly fragmented. Very importantly, the common first year for all students at UT also serves for knowledge and skills equalisation (levelling), as well as for community building.

The process of selecting students to study tracks (i.e. second-year universities) involves several stages and provides comprehensive information and guidance for the final decision making. At the beginning of the first year, students are first introduced to the study tracks through the resources available on the programme’s website and additional explanations from the academic coordinator. Throughout the first semester, students attend various information sessions including meetings with professors from second-year universities and video conferences with second-year students and alumni. These events offer insights into the specifics of each study track, academic life and practical experiences, varying from study-related questions on course contents to practicalities and logistics, such as where to find a cheap bicycle. The process culminates in a final preference survey at the end of the first semester (conducted at the yearly Winter School, see below), where students rank their study track choices based on the insights they have gained by that point. Importantly, throughout the years, it has been possible to assign the study tracks of their first preference to all students!

### Winter School

A key integration element to guarantee a cohesive master’s programme is the annual Winter School (WS) which provides a vital contribution to the “EACH community”. The Winter School is alternately hosted in the countries of the four EACH partner universities and involves both first- and second-year students, as well as alumni. It takes place yearly during 1 week in the second half of January. The travel and accommodation related to the Winter school are free for students and are funded by the *Erasmus Mundus* grant. The mini-courses delivered at the WS are academically inspiring regardless of the students’ prior experience and knowledge. Importantly, the WS also serves as a mid-term evaluation and provides networking opportunities for the first- and second-year students. It is at the WS that the first-year students are selected for their second-year universities. At the WS, after listening to the presentations of the second-year students (about their thesis work topics and the practicalities of life at second-year universities), the first-year students submit their study track preferences. The consortium committee then assigns the first-year students to study tracks, trying to maximally take into account their preferences. The students’ choices of second-year universities are typically unevenly distributed but we have some flexibility in accommodation to uneven distribution, so that up to now we have managed to assign all students to the second-year universities of their first choice. In addition, the WS contributes to the regular physical networking of partner universities and the building of relationships between key personnel.

### Success metrics

Employability of the graduates has been one of the main concerns from the very beginning of the development of the EACH programme. The learning outcomes of the programme are rooted in the Dublin descriptors, a set of five generic categories of competencies that the students need to acquire upon completion of the study programme [5], and directly address the needs of personnel (the scope of skills and expertise) in industry and laboratories as well as in private and public institutions.

It seems that the efforts related to employability have been fruitful. Namely, just in a few months (!) after graduation, 119 out of 131 graduates (joint numbers of graduates of the intakes 2015–2022, i.e. 91%) had secured, either a job or a PhD position. The remaining 9% typically found a position somewhat later. Among all graduates, 57% continued their career in academia pursuing a PhD and 43% acquired an industry-based job. The graduates from the first EACH intakes who went on to PhD studies have by now mostly obtained their PhD degree and their most frequent job description is R&D scientist or engineer at a company in the field of chemical, pharmaceutical, materials, etc. The employability statistics support the hypothesis that EACH graduates acquire relevant field-specific knowledge, professional skills, and relevant general competencies expected from an analytical chemist suitable for the current job market. A good overview of the positions held by our graduates is available in the “Career outlook” section of the EACH website. After graduation, most of our alumni have remained in the European Union to pursue a successful career. This is also illustrated by the comprehensive data published in the “Career outlook” section of our webpage. Still, there are also alumni who have returned to their home countries and are applying their new knowledge and skills to make an impact nationally.

An additional useful success metric of the programme is the dropout rate. Throughout the eight student intakes, out of the 137 admitted students, only 4 (3%) have dropped out (currently, 2 students are on extended study-time). During the most recent intakes (2021–2022), there have been no dropouts, and during the previous intake period 2018–2020 out of the 52 admitted students, only one (2%) dropped out. On the one hand, this indicates that the programme is well-structured and organised; on the other hand, it also reflects that the students joining the EACH programme are highly motivated.

### Student feedback

Feedback from the students is probably the best indicator of where the EACH programme stands today. Student feedback is extensive and is actively implemented in shaping the programme.

One of the most important (and recurring) insights from the students is that the quality of teaching is very high and demanding at the same time. Students find that the required workload matches well the volume of the academic credits they earn. Students have reported that the multi-step guidance for the study track selection indicates a systematic and well-integrated approach to planning mobility within the programme. There is a clear indication that the bridging/preparatory courses during the first year of the programme are necessary. Students have expressed their interest in advancing their generic skills and this has been addressed in the programme (data handling, entrepreneurship, programming, etc.).

The students have also praised the flexibility of the programme, e.g. the possibility to take introductory language courses of the languages of the second-year countries during the first semester or during the second semester. This also makes the students’ choice of study tracks more flexible. It frequently happens that the study track preference of students changes during the first semester, and therefore, a language course thought to be useful at the start of the studies may not be relevant by the end of the first semester.

Another important student feedback is that the mentorship received from professors who are well-known in their fields has been instrumental for them in guiding their career paths. This highlights that the EACH programme has built a good reputation in the field of analytical chemistry and is providing the students and alumni with an effective network and community for their future careers.

## Impact of the EACH programme on industry and academia

### Impact on the partner universities

The EACH programme is one of only two *Erasmus Mundus* Joint Master’s Degree (EMJMD) programme where an Estonian university is in the role of the coordinating institution (Estonian universities are participating in more JMD programmes, but not as coordinators). The current successful participation in the joint master’s degree programme proves the high academic potential of the participating universities. It has substantially increased the number of highly motivated students within all partner universities. In the case of UCBL, the programme helped to reinforce the links with industrial partners, as the industry in the Lyon area is able to recruit several EACH students every year. In return, the students can easily find opportunities for training as well as for jobs.

The increased numbers of students evidence that having combined the strengths of the four universities and their labour market partners has enabled them to attract international students whom any of the individual partners would not have been able to attract.

In addition, the EACH programme has been a role model for other emerging consortia at the four partner universities and has offered support and guidance when other EMJMD programmes have been established at the partner universities. For example, ÅAU is now coordinating an Erasmus Mundus Masters programme in IT (EDISS, https://www.master-ediss.eu/). Expertise in analytical chemistry combined with IT may further increase the impact of the EACH programme.

### Stakeholder feedback

The EACH programme has been designed in close collaboration with stakeholders (laboratories and industries), i.e. the likely future employers of the EACH alumni. Hence, the associated partners from industry and laboratories play an important role in providing EACH students with knowledge and skills, as well as influencing the learning outcomes. They also directly contribute to achieving the learning outcomes by providing internship possibilities and master’s thesis projects. The modes of collaboration are highly diverse, involving meetings, e-mail discussions, joint supervision of students, joint participation in defence committees, etc.

The EACH alumni are sought after, which is evidenced both by the aforementioned employability statistics and by the fact that the academic leaders of the programme increasingly receive job and PhD position offers with requests to distribute them to the EACH students and alumni.

The EACH programme has initiated a body called the External Advisory Council (EAC), composed of representatives of universities, professional bodies, and international organisations related to analytical chemistry. The main purpose of EAC is quality evaluation of the EACH programme and giving guidance on programme development. The quality evaluation is carried out every 3 years. The most recent EAC evaluation report is available from the “Consortium” section of the EACH website.

### Alumni success stories

Numerous testimonials from the alumni are available in the “What our students say?” and “Career outlook” sections of the EACH website. After graduation, the diploma is highly valued by potential employers. Some EACH graduates have even said that, due to several competing job options, it was challenging to choose between several opportunities, both in the industry and in academia. Some have praised the diverse international exposure across universities in Estonia, Finland, France, and Sweden, enriching their cultural and academic experiences. The supportive academic staff, access to cutting-edge technology, and industry-relevant projects are frequently cited as transformative for students’ careers.

Overall, alumni are pursuing PhD programmes, work in international companies, or take up positions in regulatory bodies, attributing their success to the comprehensive education they received. Some examples of their feedback regarding their future careers are presented here (some of them in a shortened version, the full testimonials are on the web).

Yu-Yen Ting (graduated of intake 2020): “The most valuable thing EACH programme provided was the opportunity for doing an internship … With this internship experience and the knowledge gained from EACH, I successfully secured my future position as an R&D engineer at a world-leading passive component manufacturer before graduation.”

Anh Hai Vu (graduate of intake 2019): “… what I have learned from EACH already enabled me to secure a PhD position in the field of biochemistry before my graduation. This is proving that the knowledge and the skills that the EACH programme equipped us with could open up career opportunities not only in the analytical chemistry area, but also in other fields of science …”.

Luca Guagneli (graduate of intake 2019): “*…* The ÅAU study track gave me a comprehensive expertise in the field of electrochemistry and sensors that was a strong base for my current position in the R&D field, which was secured and commenced before my graduation from EACH.”

Michal Kaczmarek (graduate of intake 2019): “In addition, EACH, bringing so many backgrounds across the world, teaches the importance of networking, understanding other cultures and different viewpoints”.

Helmi-Ulrika Gomankova (née Kirm; graduate of intake 2018): “The programme’s reach does not stop at Chemistry – graduating during a pandemic was difficult, yet I managed to start my career at a microbiology lab, proving that the EACH programme is appreciated beyond the chemistry field as well.”

### Academic contributions

The education of the EACH programme is rooted in science and the master’s theses comprise scientific or applied research projects. Exposure to different academic environments and advanced laboratory facilities fosters innovation and enhances expertise in analytical chemistry. This dynamic environment not only benefits the students but also strengthens the scientific contributions of the host institutions, driving progress in the field. Although a master’s thesis is not required to result in a scientific publication, in several cases, the research carried out by EACH students resulted in scientific publications in high-ranking international peer-reviewed journals [6–14].

## Evolution and expansion of the curriculum and new trends

### Curriculum evolution

The EACH academic board is constantly reviewing and improving the EACH curriculum. A main force driving the changes are the students and alumni.

The main admission requirement in terms of prior chemistry knowledge is that at least 60 ECTS of chemistry has been studied in the previous study level. This requirement is met in essentially all chemistry and chemical engineering BSc programmes, also in most pharmacy, biotechnology, etc. programmes. As a result, students of different backgrounds are admitted to the EACH programme. Thus, levelling/bridging activities are highly important during the first study year. The bridging activities in the EACH programme have constantly evolved as we have gained more experience. Right now, they consist of the following: a levelling course on calculations in analytical chemistry (1 ECTS), the beginners’ lab course (3 ECTS), and (linked to both of these courses) face-to-face consultations with students who need more help. These courses and consultations take place during semester 1. They allow the teaching staff to identify topics where students need additional guidance and support so that it can be taken into account in subsequent courses. It has turned out that newly admitted students often need help in topics related to basic calculations in analytical chemistry (preparing and diluting solutions, etc.) and basic lab skills (weighing, pipetting, etc.). We are convinced that these bridging activities are among the main reasons for the low dropout number of the programme (see above).

In order to meet the expectations of society and the job market, as well as at the request of students and alumni, elective courses are constantly added to the programme. Thus, in addition to different analytical chemistry courses, students can now choose from courses on entrepreneurship, forensics, programming, and big data handling during the first study year. At second-year universities, the courses are also constantly evolving, specifically in their fields of speciality. Particularly interesting in this respect is the possibility offered jointly by the ÅAU and the University of Turku (UTU), so that EACH students can take elective courses also from UTU. Collaboration between ÅAU and UTU is beneficial as ÅAU and UTU are located in the same campus area, where ÅAU and UTU jointly host the Turku Centre for Chemical and Molecular Analytics (CCMA, https://ccma.fi/), which is one of the best-equipped NMR/MS facilities in Finland.

An important activity that is constantly in process is fine-tuning the first-year courses in such a way that they would best support the specialisation during the second year. First-year courses contain practical examples or case studies related to the thesis topics offered to students during their second year and dedicated elective courses are offered to support second-year studies (e.g., “Chemometrics” for UCBL, “Liquid Chromatography and Mass Spectrometry” for UU, and “Introduction to Electroanalysis” for ÅAU).

Another important set of factors directing the development of the programme are the emerging areas where analytical chemists are much needed: mitigating climate change, reducing pollution, developing renewable energy, sustainable industry and food sources, healthcare and waste valorisation. We are constantly intertwining these topics into the EACH programme. Automatic monitoring of the environment (oceans, atmosphere, etc.) is a good example. Such monitoring is key to developing environmental protection, mitigating climate change and ensuring a sustainable future. Such monitoring, e.g. the ARGO international oceanographic monitoring programme, has been well on its way for decades [15]. The close to 4000 floats, spread all over the world’s oceans, monitor several parameters (temperature, salinity, pH, dissolved oxygen, etc.). However, many more would be desired, such as continuous monitoring of nitrate and phosphate in coastal areas, lakes, and rivers to mitigate eutrophication. Emerging pollutants, such as polyfluorinated organic substances, are persistent and toxic compounds that tend to accumulate in the food chains. Other examples include pharmaceuticals and hormones that can be detrimental even at extremely low concentrations when released into natural waters. The reason they are not continuously monitored is simple: unavailability of reliable sensors. Another rapidly developing field is the use of wearable chemical sensors and biosensors for on-body monitoring of biomarkers such as glucose, lactate, and electrolytes [16]. Again, the currently limited extent of using them is due to the unavailability of suitable sensors. The field of sensors is covered by Åbo Akademi University where students study and do research at the forefront of modern sensor design and fabrication and analysis of sensor measurement data. In the healthcare and biomedical sector, a similar fast progression in multi-omics technologies takes place, to provide precision medicine for individual patients. At Uppsala University (UU), the focus is both on targeted and untargeted mass spectrometry-driven metabolomics and proteomics as very powerful tools. The technologies are also generating massive complex data amounts and hence why also bioinformatics and biostatistics are addressed in the programme. At UCBL, the focus is on online analysis for chemical plants. Knowing the exact composition of the substances circulating in an industrial plant is critical information for companies in the energy, environmental, chemical, and even food industries. With these kinds of data, companies can control costs, respond efficiently to emergencies, and adjust production parameters. Current technologies are now reaching their limits and new types of analysers, small, fast, and reliable, are required.

Visiting scholars are invited every year to cover some of the new and emerging fields and topics in analytical chemistry and related areas. They contribute by giving students a broader view of analytical chemistry as a discipline, specifically from the “real-life” perspective. Some examples are (short) courses on online process control in industry, advanced fluorescence spectroscopy, forensic science, environmental monitoring, cutting-edge sensor technology, etc. Most importantly, thanks to the visiting scholars, the students get an extensive overview of the actual contemporary needs of the chemical industry, as well as biotech start-ups.

Modern analytical chemistry relies heavily on instruments and instruments are constantly developing. Thus, the student lab infrastructure at the four EACH partner universities is constantly evolving and together with it also the lab experiments that students perform. This ranges from quantification of pesticide residue in tangerines with LC–MS to quantitative ATR-FT-IR analysis with multivariate statistics for quantification during the first year. Notable developments take place also at second-year universities. The labs at ÅAU have been equipped with new potentiostats that are essential tools for teaching and research related to sensor fabrication and characterisation, including the development of novel signal transduction mechanisms for ion sensors. Real sensor prototypes, such as solid-contact ion-selective electrodes, solid-state reference electrodes, gas sensors, and biosensors, are built as part of master’s thesis projects. At UU, there is a constant reinvestment in especially devices for sample preparation and high-resolution LC–MS/MS for both research and education. Some of the infrastructure resources are also co-funded by the dedicated quality funding support from the University. UCBL teaching facilities invest a lot in the lab dedicated to analytical science, with NMR, ICP-MS, and GCxGC-TOF instruments being the latest acquisitions. UCBL has also implemented a paradigm shift in teaching instrumental analysis, allowing students to design and develop their own very low-cost instruments and use them as part of their science learning process. Through team projects (one EACH student, one Physics student, and one Electronics student), the students are also equipped with cross-disciplinary communication skills ranging from using digital technology to entrepreneurial development. Last year, a project with a strong societal impact “Sorting plastic sheets” was carried out.

The EACH programme has integrated two massive open online courses (MOOCs) into the curriculum—“Estimation of Measurement Uncertainty in Chemical Analysis” (https://sisu.ut.ee/measurement/uncertainty [17]) and “Validation of LC–MS methods” (https://sisu.ut.ee/lcms_method_validation/). These courses are available annually. Importantly, a number of EACH students who participate in the MOOCs are from the second year, meaning that all EACH study tracks benefit. Having MOOCs (or other online courses) integrated into the programme provides the students, among other things, with the skills needed for life-long learning and enhances their IT competence. On the other hand, including our lecturers in the development of MOOCs also elevates their IT and hybrid teaching/learning skills.

We have discovered that in addition to being a useful component of the programme, MOOCs have worked efficiently as a promotion tool. Up to now, we have had overall more than 12,000 participants in our two MOOCs from over 150 countries and a number of EACH students first learned about our programme from the MOOCs. This means that a very large number of analytical chemistry professionals in the world are aware of the EACH programme.

### International cooperation

The EACH programme is connected to several international chemistry organisations and our students are constant participants in international conferences and MSC Summer Schools.

The staff of the consortium, namely UT, Estonian Environmental Research Centre (EKUK), and Research Institute of Sweden (RISE), participate in the work of Eurachem (https://www.eurachem.org/), the biggest and most influential analytical chemistry organisations in Europe and one of the most influential in the world. UT was the organiser of the Eurachem 2019 General Assembly and the accompanying workshop in Tartu (http://eurachem2019.akki.ut.ee/), and at that event, there was a special session on education where the EACH programme received significant attention from an audience of European “top players” in analytical chemistry. Although Eurachem is formally a European organisation, the event attracted a large number of non-European participants.

In 2023, EACH students had the opportunity to participate in the Ecobalt2023 conference in Tallinn (https://ecobalt2023.kbfi.ee/). Ecobalt is organised once every 2 years and is meant for environmental scientists, ecologists, analytical and organic chemists, material scientists, toxicologists, and risk assessors to discuss the current topics related to the environment. Ecobalt2023 brought together current EACH students and many EACH alumni who are either working or doing their PhD degrees in neighbouring countries. EACH students have been invited to attend both international and national MS conferences and have had the opportunity to present their research projects while building their networks. As part of their master’s programme in France, EACH students participate in a professional event “Salon de l’Analyse Industrielle” (Industrial Analysis) where they can attend conferences and workshops as well as directly interact with instrument providers and users.

EACH students can participate in the summer school of the Measurement Science in Chemistry (MSC) consortium (http://www.msc-euromaster.eu/) [18]. This summer school is considered by many to be the best in Europe in the field of chemical measurement science/analytical chemistry and attracts numerous students from the Erasmus + partner countries, most notably from North Africa and Southeast Asia. The key activity at the summer school is a contest of student teams—acting as small companies offering analytical services—in setting up and validating an analytical method for a “customer” (played by one of the teachers)and thereafter carrying out real analysis of the customer’s sample. The summer school is very intensive, and the participants need to use all their knowledge and skills for satisfying the demanding customer.

## Collaborations and industry partnerships

### New partnerships

The EACH programme has numerous educational and non-educational associated partners all over the world. In the current edition of the programme, there are 22 industry and laboratory partners, out of which 7 are new in comparison to the last grant period (2018–2022). In addition to industry partners, there are also 11 educational partners (with 2 being new). The full list of partners is available in the “Associated partners” section of the EACH website. Importantly, the associated partners are not only from the countries of the partner universities but altogether from 12 countries, showing a broad interest in such a programme. In addition to industry partners, there are also 11 educational partners (with 2 being new) and these truly cover the world, since the associated partners are from North America to China, with Spain and India in between, to name a few.

### Internships and real-world application

Right now, over 50 different companies or institutions offer internship opportunities to EACH students. The EACH students have carried out their internships in different countries—most often in Estonia, Finland, and France but also in Latvia, Malta, Austria, Spain, etc. The number of partners offering internships has steadily increased over the years as there have been more companies joining. Activities of the companies or institutions include state agencies, private companies, research institutions, and many more, covering fields from supramolecular chemistry to food analysis. During an internship placement, students spend approximately 2 months in the company or institution working on everyday problems that are encountered there under the supervision of the staff members.

Academic coordinators of the four partners jointly organise student internships by constantly looking for organisations and making agreements in their respective countries. This is beneficial not only to the students but also to the consortium because it helps to build relationships with industry and research institutes outside academia.

The average distribution of students among the different internship providers over the years is approximately as follows: one-third do their internship at the industrial partners in the Lyon area, one-third do the internship at other associated partners, and one-third of students find their internship place outside the EACH consortium.

Importantly, many practitioners from the industry have participated in the programme as lecturers and supervisors of master’s theses: Franck Baco-Antoniali (Axel'One, a collaborative platform, developing analysis solutions for industry), Noémie Caillol (Axel'One), Marion Lacoue-Negre (IFPEN, an energy company), David Speybrouck (Servier, a pharmaceutical company), David Chiche (IFPEN), Marlene Peyrillous (Elkem, polymers/chemical industry), and others. They are from the industrial-associated partners in the Lyon area and participate in the Industrial Analysis study track. Their contribution is indispensable to the programme.

## Challenges and adaptations

### COVID-19 impacts

The main result of the COVID-19 pandemic has been that a lot of teaching in the EACH programme is also now performed in hybrid mode, e.g. lectures run in lecture halls and online in parallel, enabling the participation of students who for some reason (e.g. illness) cannot attend the lecture physically and making lectures available for both first- and second-year students. Additionally, recordings of classes are available. This is especially helpful for attracting external scholars who otherwise would not be able to come because of being highly occupied. The availability of online/hybrid teaching also puts less pressure on the students if there are problems arriving at their study locations on time or if there is a need for emergency travelling back to their home countries far away.

### Student mobility and international cooperation

**Fig. 2:**
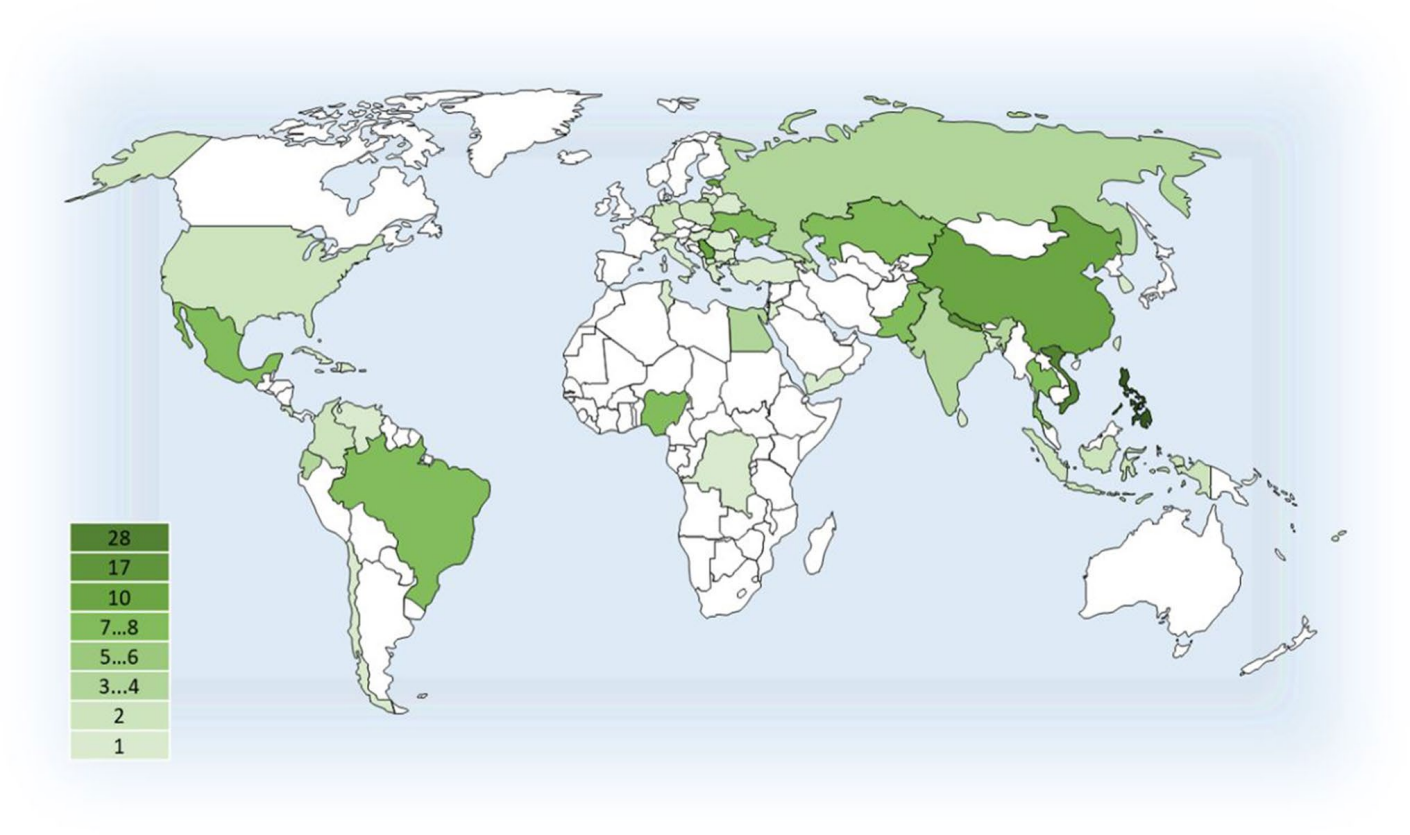
Countries of origin of the current EACH students and graduates

The overall number of EACH students (including those who are studying right now) since the start of the programme (i.e. ten intakes) has been 173. The current students and graduates of the EACH programme originate from altogether 51 countries (Fig. 2), the largest number being from the Philippines (28), followed by Vietnam (17) and Nepal (10). The number of students from the EU countries has been rather low and we do not have an explanation for that.

However, the situation in the world right now is not as welcoming to student mobility as it was in the past, due to geopolitical conflicts, wars, and rising anti-immigrant sentiment. Nevertheless, the EACH programme is special in a way that being a chemistry programme, it crosses borders and currently has the highest number of students of any chemistry-related master’s programme at UT and contributes also substantially to student numbers in ÅAU, UCBL, and UU. In addition, EACH programme provides international study experience for the local students—both in terms of classroom work and thesis work, since research groups also become more international.

Further, the EACH student community provides a convenient “academic home” for other visiting international students who spend a semester or two at UT. Typically, such students are left pretty much on their own, but thanks to the EACH programme, they are within a welcoming community of international students. Students have also reported in their feedback that the programme offers many options for practising teamwork in multinational and multicultural groups and the experiences gained are essential in their future careers.

Similar observations can be made for UU, UCBL, and ÅAU. At all three second-year universities, participation in EACH has enhanced the quality and international outreach of teaching. For example, the existence of EACH has significantly enforced teaching in English at UCBL, where before EACH analytical chemistry teaching was almost exclusively in French. The industry is now well aware of the EACH programme and EACH students can easily find opportunities for training as well as for jobs. All in all, EACH has been a role model for other joint international programmes at partner universities and contributed to the internationalisation of the related departments.

## Future directions and vision for the future

With constant progress in the field of analytical chemistry, there is a clear vision for the future which holds continuous evolvement and goes along with the needs of society. There needs to be room for advanced technologies like AI, machine learning, and virtual reality for hands-on and remote learning while fostering interdisciplinary collaboration with fields like biology, data science, and engineering. The programme needs to meet the expectations of the students as well as the job market, meaning there is a continuous need for real-world industry applications, but on the other hand, also personalised, modular learning paths. Where possible, the EACH programme can also nurture innovation and entrepreneurship by encouraging curiosity and where possible, integrate patent and IP education to encourage turning scientific discoveries into impactful solutions.

One of the biggest future initiatives, right now underway, is developing EACH into a pan-European Analytical Chemistry Hub. This means involving universities from different European University Alliances (Enlight, ARQS, CharmEU, ForTHEM, etc.) with the aim of developing a shared European competence framework, aligning curricula with competence framework, and creating resources with a European dimension. With such an analytical chemistry education initiative—a large-scale exchange of ideas and information between high education establishments, public bodies, and industries—the EACH programme will be established as a pan-European role model of excellence in analytical chemistry teaching via initiatives that are new to the field. This will cover both new activities for the students and bring together teaching professionals to build a network for spreading the knowledge of teaching analytical chemistry.

The joint nature of the programme is constantly deepening, and as a result, the consortium has decided that as a next step, the programme will implement a joint degree, which will be awarded to the next intake of students.

A 2-year programme, while limited in time, can provide a strong foundation for students by focusing on fundamental knowledge, real-world experiences, and exposure to key technologies. Though it may not cover every aspect of analytical chemistry or its evolving interdisciplinary applications, it can equip students with critical thinking, problem-solving skills, and an adaptable mindset needed for lifelong learning. By emphasising the fundamentals, encouraging curiosity, and offering pathways for continuous development, the EACH programme can effectively nurture students’ ability to grow and thrive in a rapidly advancing field, preparing them to build upon their knowledge throughout their careers.

## References

[1] Salzer R, Hrastelj N, Smith A. European Employment Survey for Chemists (ESEC3) Careers of Chemistry Graduates in Europe. Chemistry A European J. 2024;30:e202401223. 10.1002/chem.202401223. 10.1002/chem.202401223 38752275

[2] Salzer R, Cole-Hamilton D, Hrastelj N, Vilela B. Employment and Careers of European Chemists (ESEC2). Chemistry A European J. 2018;24:17370–88. 10.1002/chem.201804764. 10.1002/chem.201804764 PMC6587813 30457685

[3] Grinias JP, Smith TI, Kovarik ML. LCGC and ACS Subdivision on Chromatography and Separations Chemistry Survey 2023: What Skills Do New Analytical Chemistry Employees Need? LCGC N Am. 2023;284–286. 10.56530/lcgc.na.uc7884m3.

[4] Leito I, Teearu A, Bobacka J, Randon J, Bergquist J. EACH (Excellence in Analytical Chemistry), an Erasmus Mundus Joint Programme: progress and success. Anal Bioanal Chem. 2019;411:5913–21. 10.1007/s00216-019-01988-8. 31392438 10.1007/s00216-019-01988-8

[5] Joint Quality Initiative informal group. Shared ‘Dublin’ descriptors for Short Cycle, First Cycle, Second Cycle and Third Cycle Awards. Dublin; 2004. https://www.hrk.de/fileadmin/redaktion/hrk/02-Dokumente/02-03-Studium/02-03-02-Qualifikationsrahmen/dublin_descriptors-1.pdf. Accesed 17 Jan 2025

[6] Mandjoukov B, Lindfors T. Planar, low-cost, flexible, and fully laminated graphene paper pseudo-reference and potassium-selective electrodes. Sens Actuators, B Chem. 2024;403:135190. 10.1016/j.snb.2023.135190.

[7] Manandhar S, Yrjänä V, Leito I, Bobacka J. Determination of benzoate in cranberry and lingonberry by using a solid-contact benzoate-selective electrode. Talanta. 2024;274:125996. 10.1016/j.talanta.2024.125996. 38574535 10.1016/j.talanta.2024.125996

[8] Blidi S, Granholm K, Sokalski T, Mousavi Z, Lewenstam A, Leito I, Bobacka J. Long-Time Evaluation of Solid-State Composite Reference Electrodes. Membranes. 2022;12:569. 10.3390/membranes12060569. 35736276 10.3390/membranes12060569 PMC9230823

[9] Palmblad M, Asein E, Bergman NP, Ivanova A, Ramasauskas L, Reyes HM, Ruchti S, Soto-Jácome L, Bergquist J. Semantic Annotation of Experimental Methods in Analytical Chemistry. Anal Chem. 2022;94:15464–71. 10.1021/acs.analchem.2c03565. 36281827 10.1021/acs.analchem.2c03565 PMC9647698

[10] Guagneli L, Mousavi Z, Sokalski T, Leito I, Bobacka J. Novel design of a planar flow-through potentiometric sensor. J Electroanal Chem. 2022;923:116785. 10.1016/j.jelechem.2022.116785.

[11] Leesment A, Selberg S, Tammiste M, Vu AH, Nguyen TH, Taylor-King L, Leito I. Quantifying Acidity in Heterogeneous Systems: Biphasic p *K*_a_ Values. Anal Chem. 2022;94:4059–64. 10.1021/acs.analchem.1c05510. 35195999 10.1021/acs.analchem.1c05510

[12] Manandhar S, Sjöholm E, Bobacka J, Rosenholm JM, Bansal KK. Polymer-Drug Conjugates as Nanotheranostic Agents. JNT. 2021;2:63–81. 10.3390/jnt2010005.

[13] Liu H, Raffin G, Trutt G, Randon J. Is vacuum ultraviolet detector a concentration or a mass dependent detector? J Chromatogr A. 2017;1530:171–5. 10.1016/j.chroma.2017.11.028. 29157607 10.1016/j.chroma.2017.11.028

[14] Neupane R, Bergquist J. Analytical techniques for the characterization of Antibody Drug Conjugates: Challenges and prospects. Eur J Mass Spectrom (Chichester). 2017;23:417–26. 10.1177/1469066717733919. 29183195 10.1177/1469066717733919

[15] Riser SC, Freeland HJ, Roemmich D, Wijffels S, Troisi A, Belbéoch M, Gilbert D, Xu J, Pouliquen S, Thresher A, Le Traon P-Y, Maze G, Klein B, Ravichandran M, Grant F, Poulain P-M, Suga T, Lim B, Sterl A, Sutton P, Mork K-A, Vélez-Belchí PJ, Ansorge I, King B, Turton J, Baringer M, Jayne SR. Fifteen years of ocean observations with the global Argo array. Nat Clim Chang. 2016;6:145–53. 10.1038/nclimate2872.

[16] Boeva Z, Mousavi Z, Sokalski T, Bobacka J. Recent trends in non-invasive on-body chemical sensing. TrAC, Trends Anal Chem. 2024;172:117542. 10.1016/j.trac.2024.117542.

[17] Leito I, Helm I, Jalukse L. Using MOOCs for teaching analytical chemistry: experience at University of Tartu. Anal Bioanal Chem. 2015;407:1277–81. 10.1007/s00216-014-8399-y. 25577351 10.1007/s00216-014-8399-y

[18] Taylor PDP, Barałkiewicz D, Bettencourt Da Silva R, BrodnjakVončina D, Bulska E, Camoes MF, Dobrowolski R, Elskens M, Leito I, Majcen NH, Mandjukov P, McCourt J, Randon J, Perämäki P. A summer school where master students learn the skills needed to work in an accredited analytical laboratory. Anal Bioanal Chem. 2015;407:6899–907. 10.1007/s00216-015-8804-1. 26187319 10.1007/s00216-015-8804-1

